# Data on the daily electricity load profile and solar photovoltaic (PV) system components for residential buildings in Lagos, Nigeria

**DOI:** 10.1016/j.dib.2020.105531

**Published:** 2020-04-13

**Authors:** Kevin Enongene Enongene, Fonbeyin Henry Abanda, Iduh Jonathan Joseph Otene, Sheila Ifeakarochukwu Obi, Chioma Okafor

**Affiliations:** a FOKABS INC, 955 Rotary Way, K1T 0L2 Ottawa ON, Canada; b School of the Built Environment, Faculty of Technology, Design and Environment, Oxford Brookes University, Oxford OX3 0BP, UK; c Institute of Agriculture and Environment, School of Sciences, Massey University, Palmerston North 4474, New Zealand; d Department of Electrical/Electronics Engineering Technology, Akanu Ibiam Federal Polytechnic Unwana, PMB 1007, Ebonyi State, Nigeria; e Department of Soil Science, University of Nigeria Nsukka 410001, Nigeria

**Keywords:** Energy, Nigeria, Renewable energy, Photovoltaic, Residential buildings

## Abstract

This article contains the average daily electric load profile (for 24 h of the day) for the five categories of residential buildings (duplex, single family bungalow, traditional court yard, flat/apartment dwelling and ‘face-me-I-face-you’) in three Local Government Areas (LGAs) of the state of Lagos, Nigeria. In each of the LGAs, 10 buildings per residential building type were surveyed for the collection of data with the aid of a questionnaire. In each surveyed household, a household member completed the energy audit section of the questionnaire with the assistance of the questionnaire administrator while the section of the questionnaire designed as a time-of-use diary was left with the household for completion. For each building surveyed, the data retrieved from the completed time-of-use diary was used in Microsoft Excel for computing the hourly electricity load profile for the seven days of the week. In order to obtain the hourly energy load (in watts) for each building, the power rating of the appliances used during each of the 24 h of the day was summed and the result in watts was converted to kWh by dividing by 1000. Each dwelling's daily load profile was obtained as an average of the load profile for the seven days of the week. The article as well provides data on the solar photovoltaic systems’ components designed to supply electricity to the building and the levelized cost of electricity (LCOE) of the systems for the base case scenario and different sensitivity cases obtained from simulations using HOMER Pro. The load profile data provided in this article can be reused by other researchers in the design of solar photovoltaic systems for residential buildings.

**Specifications Table**

**Table:** 

Subject	Energy
Specific subject area	Renewable Energy, Sustainability and the Environment
Type of data	Table
How data were acquired	Survey (with the aid of a questionnaire) Software (HOMER Pro)
Data format	Raw
Parameters for data collection	In collecting the data, the principle of free, prior and informed consent was respected. Prior to questionnaire administration and data collection, the objective of the research was well-explained to the respondents and their consent for participating in the research sought. Data collection was limited to those respondents who gave their consent to participate in the research.
Description of data collection	Data was obtained through household surveys with the aid of a questionnaire and modelling using the HOMER Pro software
Data source location	Lagos (Latitude 6°27′14″N and Longitude: 3°23′40″ E), Nigeria
Data accessibility	Data is with this article (in the Appendix)
Related research article	The potential of solar photovoltaic systems for residential homes in Lagos city of Nigeria. Journal of Environmental Management (https://doi.org/10.1016/j.jenvman.2019.04.039).

## Value of the data

• The data provides an estimate of residential electricity loads for different categories of buildings in Nigeria which could also be adopted for different developing countries.
• The load profile data could be used by other researchers interested in designing off-grid renewable energy systems for residential buildings.
• The data on the LCOE of systems designed for the different building categories could be used as a benchmark for further research in renewable energy applications in residential buildings.

## 1 Data description

This article includes data on the daily electric load profiles and corresponding solar PV components for the different categories of residential buildings in Lagos, Nigeria. Table A1 (Appendix A) presents the minimum and maximum hourly electric loads for each category of building per LGA. Tables B2 and B3 (Appendix B) presents the effects of the variation of minimum battery state of charge and capacity shortage on PV system components and LCOE for the systems designed for the maximum (Table B2) and minimum (Table B3) electric loads for the different categories of building per LGA. Tables C4 and C5 (Appendix C) Portrays the effect of the variation of discount rate, inflation rate, PV lifetime and battery state of charge on the LCOE of the PV systems for the maximum (Table C4) and minimum load (Table C5) of buildings.

## 2 Experimental design, materials, and methods

Residential buildings from three Local Government Areas (LGAs): Kosofe, Oshodi and Alimosho in Lagos Metropolitan Area, Lagos State of Nigeria were surveyed. The survey was conducted using a structured questionnaire and entailed purposive sampling. Lagos is divided into five Administrative Divisions (Lagos, Epe, Badagry, Ikorodu and Ikeja) which are further divided into 20 Local Government Areas (LGAs) and 37 Local Council Development Areas (LCDAs). In each of the LGAs, 10 buildings per residential building type Nigeria (duplex, single family bungalow, traditional court yard, flat/apartment dwelling and ‘face-me-I-face-you’) as identified by Jiboye [1] were surveyed. In each surveyed household, a household member completed the energy audit section of the questionnaire with the assistance of the questionnaire administrator while the section of the questionnaire designed as a time-of-use diary was left with the household for completion. For each building surveyed, the data retrieved from the completed time-of-use diary was used in Microsoft Excel for computing the hourly electricity load profile for the seven days of the week. In order to obtain the hourly energy load (in watts) for each building, the power rating of the appliances used during each of the 24 h of the day was summed and the resulting value converted to kWh by dividing by 1000. Each dwelling's daily load profile was obtained as an average of the load profile for the seven days of the week.

For each building type per LGA, load profiles representing the maximum and minimum building load were used in the HOMER Pro software for modelling the PV systems. The software modelled the system configuration's behaviour for each hour of the year so as to determine the life cycle cost and the technical feasibility of the system. This involves optimization of the system through the simulation of several system configurations with the aim of identifying the system that meets the technical constraints at the lowest life cycle cost. The calculation for the base case scenario was conducted as per the following parameters: 2% and 5% inflation rate and discount rate respectively, a 25-year PV-system lifetime, maximum annual capacity shortage of 0% and 40% minimum battery state of charge (SOC).

Sensitivity analysis was conducted with HOMER Pro based on five variables: inflation and discount rates, lifetime of PV system, maximum annual capacity shortage and minimum battery state of charge. The sensitivity analysis was conducted in order to investigate the effect of the variables on the LCOE of the systems. Table 1 presents the sensitivity parameters used in the analysis.

**Table 1:** Sensitivity parameters used in performing the sensitivity analysis with HOMER Pro [2].

Sensitivity variable	Base case	Sensitivity case(s)
PV-system lifetime	25 years	30 years and 20 years
Minimum battery SOC	40%	30%
Inflation rate	2%	5%
Maximum annual capacity shortage	0%	15%, 10% and 5%
Discount rate	5%	10%

## Figures and Tables

**Table A1:** Minimum and maximum loads of building types across the different LGAs.

LGA	Hour of the day (hourly load data in kWh) – MINIMUM LOADS																							
	0	1	2	3	4	5	6	7	8	9	10	11	12	13	14	15	16	17	18	19	20	21	22	23
Building type: Single Family Bungalow																								
Alimosho	0.000	0.000	0.000	0.000	0.000	0.000	0.000	0.000	0.000	0.000	0.000	0.000	0.000	0.255	1.056	1.341	1.341	0.833	0.926	0.667	0.573	0.573	0.542	0.000
Oshodi	0.160	0.160	0.000	0.000	0.000	0.000	0.000	0.000	0.000	0.000	0.000	0.000	0.299	0.331	0.597	0.397	0.499	0.466	0.000	0.361	0.594	0.594	0.594	0.160
Kosofe	0.020	0.000	0.000	0.000	0.000	0.000	0.000	0.000	0.000	0.000	0.000	0.000	0.000	0.000	0.000	0.000	0.000	0.231	0.053	0.118	0.084	0.085	0.043	0.021
Building type: Flat Apartment																								
Alimosho	0.000	0.000	0.000	0.000	0.000	0.000	0.000	0.000	0.000	0.369	0.792	0.621	0.221	0.152	0.152	0.129	0.000	0.000	0.388	0.646	0.699	0.806	0.685	0.129
Oshodi	0.144	0.143	0.000	0.000	0.000	0.000	0.000	0.000	0.000	0.000	0.026	0.026	0.026	0.014	0.002	0.002	0.002	0.000	0.000	0.000	0.156	0.156	0.144	0.144
Kosofe	0.000	0.000	0.000	0.000	0.000	0.000	0.043	0.000	0.000	0.000	0.000	0.000	0.000	0.000	0.034	0.269	0.069	0.069	0.046	0.061	0.064	0.089	0.074	0.014
Building type: *‘Face -me -I -Face –you’*																								
Alimosho	0.000	0.000	0.000	0.000	0.000	0.000	0.000	0.000	0.000	0.431	2.331	3.424	3.424	2.729	1.404	0.000	0.000	0.000	0.000	0.000	0.000	0.000	0.000	0.000
Oshodi	0.221	0.209	0.209	0.209	0.209	0.209	0.132	0.132	0.154	0.154	0.150	0.021	0.000	0.000	0.000	0.000	0.000	0.000	0.317	0.321	0.328	0.328	0.248	0.221
Kosofe	0.000	0.000	0.012	0.012	0.012	0.000	0.029	0.029	0.000	0.000	0.000	0.000	0.000	0.029	0.029	0.029	0.000	0.000	0.000	0.043	0.043	0.043	0.000	0.000
Building type: Duplex																								
Alimosho	1.198	0.560	0.649	0.649	0.649	0.552	2.102	2.626	2.147	1.666	0.240	0.071	0.000	0.001	0.065	0.082	0.435	0.507	0.455	0.698	1.269	1.440	1.466	1.325
Oshodi	0.000	0.000	0.000	0.000	0.000	0.000	0.000	0.000	0.000	0.000	0.000	0.000	0.903	1.580	0.769	0.131	0.041	0.000	0.000	0.000	0.000	0.000	0.000	0.000
Kosofe	0.033	0.023	0.023	0.023	0.023	0.023	0.316	0.273	0.001	0.000	0.000	0.000	0.000	0.000	0.011	0.011	0.023	0.014	0.026	0.031	0.116	0.126	0.126	0.084
Building type: Traditional Court																								
Alimosho	0.000	0.029	0.029	0.029	0.029	0.029	0.000	0.107	0.148	0.096	0.136	0.119	0.135	0.148	0.107	0.107	0.032	0.079	0.066	0.135	0.081	0.041	0.029	0.000
Oshodi	0.000	0.000	0.000	0.000	0.000	0.000	0.000	0.000	0.000	0.226	0.000	0.251	0.226	0.226	0.000	0.000	0.000	0.000	0.000	0.000	0.000	0.000	0.000	0.000
Kosofe	0.011	0.023	0.034	0.046	0.046	0.023	0.000	0.000	0.000	0.000	0.000	0.000	0.000	0.000	0.000	0.000	0.000	0.000	0.000	0.000	0.057	0.057	0.057	0.000

LGA	Hour of the day (hourly load data in kWh) – MAXIMUM LOADS																							
	0	1	2	3	4	5	6	7	8	9	10	11	12	13	14	15	16	17	18	19	20	21	22	23

Building type: Single Family Bungalow																								
Kosofe	0.230	0.230	0.230	0.216	0.216	0.000	0.000	0.000	0.337	0.030	0.024	0.024	0.024	0.024	0.024	0.024	0.024	0.024	0.024	0.024	0.786	0.794	0.781	0.779
Oshodi	0.514	0.502	0.502	0.502	0.416	0.416	1.221	1.266	1.514	1.347	0.957	0.546	0.327	0.137	0.997	1.138	1.875	2.409	3.765	2.921	2.130	2.118	1.558	0.741
Alimosho	0.000	0.000	0.000	0.000	0.000	0.000	0.000	0.000	0.000	0.000	0.000	0.000	0.000	0.000	0.000	0.000	0.000	0.180	4.340	4.346	3.760	3.654	2.880	0.569
Building type: Flat Apartment																								
Kosofe	0.000	0.000	0.000	0.000	0.000	0.000	0.000	0.000	0.000	0.000	0.000	0.000	0.000	0.000	0.000	0.000	0.000	0.000	0.000	1.247	1.574	0.429	0.402	0.000
Oshodi	0.310	0.310	0.310	0.310	0.310	0.310	3.000	3.000	0.114	0.114	0.000	0.000	0.000	0.200	0.200	0.000	0.000	2.661	3.655	0.711	0.955	1.069	0.748	0.310
Alimosho	0.000	0.000	0.000	0.000	0.000	0.000	0.000	0.000	0.000	0.000	0.000	0.000	0.000	0.000	0.000	0.000	0.000	0.000	0.000	7.567	7.938	3.472	3.459	3.344
Building type: *‘Face -me –I- Face -you’*																								
Kosofe	0.100	0.011	0.011	0.011	0.011	0.011	0.000	0.000	0.000	0.000	0.000	0.000	0.000	0.000	0.000	0.000	0.000	0.000	0.023	0.884	0.485	0.485	0.249	0.225
Oshodi	0.220	0.220	0.220	0.209	0.129	0.129	0.033	0.033	32.890	0.033	0.044	0.044	0.044	0.034	0.034	0.034	0.034	0.000	0.150	0.344	0.344	0.344	0.327	0.310
Alimosho	0.000	0.000	0.000	0.000	0.000	0.000	0.000	0.000	0.000	0.000	0.143	0.431	2.508	5.705	10.282	10.288	11.888	10.734	7.876	7.876	2.848	2.052	0.853	0.000
Building type: Duplex																								
Kosofe	0.203	0.159	0.159	0.069	0.046	0.046	0.741	0.699	0.604	0.000	0.000	0.000	0.119	0.119	0.119	0.049	0.049	0.000	0.000	0.481	1.151	0.994	0.791	0.283
Oshodi	0.581	0.539	0.474	0.350	0.243	0.243	0.436	1.333	3.960	3.925	3.028	3.085	3.085	3.085	3.085	3.085	0.740	0.738	0.740	1.697	1.811	1.062	0.759	0.648
Alimosho	1.342	0.043	0.043	0.043	0.043	0.043	3.875	4.086	1.210	0.650	0.393	0.400	0.019	0.019	0.292	0.292	0.931	0.811	1.899	4.041	5.383	4.995	4.138	1.702
Building type: Traditional Court																								
Kosofe	0.000	0.000	0.000	0.000	0.000	0.000	0.000	0.000	0.000	0.000	0.000	0.000	0.000	0.000	0.000	0.000	0.000	0.000	0.014	0.277	0.302	0.113	0.051	0.000
Oshodi	0.000	0.000	0.000	0.000	0.000		0.000	0.000	0.000	0.000	0.000	0.000	1.580	1.580	0.180	0.880	1.580	1.580	0.000	0.000	0.000	0.000	0.000	0.000
Alimosho	0.263	0.000	0.000	0.000	0.000	0.000	0.000	0.000	0.000	0.000	0.000	0.441	0.591	0.591	0.806	1.594	1.709	1.807	1.954	2.011	1.943	1.874	1.616	0.838

**Table B2:** Effect of minimum battery state of charge and capacity shortage on PV system components and LCOE (maximum loads) Maximum loads.

LGA	Sensitivity value (%)	PV array (kWh)	1 kWh Lead acid battery	PV power output (kWh/year)	LCOE
**Building Type: Single family bungalow**					
Sensitivity variable: Maximum annual capacity shortage					
Kosofe	0	3	30	4194	0.508
	5	2	24	2796	0.399
	10	2	14	2796	0.385
	15	2	12	2796	0.38
Oshodi	0	3	30	4194	0.452
	5	13	86	18,174	0.312
	10	11	78	15,378	0.297
	15	10	66	13,980	0.288
Alimosho	0	15	108	20,972	0.513
	5	9	80	12,583	0.426
	10	8	68	11,185	0.412
	15	7	64	9787	0.403
Sensitivity variable: Minimum battery state of charge					
Kosofe	30%	3	26	4194	0.463
Oshodi	30%	22	115	30,756	0.416
Alimosho	30%	12	112	16,778	0.482
**Building Type: Duplex**					
Sensitivity variable: Maximum annual capacity shortage					
Kosofe	0	0.8	9	1118	0.552
	5	0.6	5	839	0.41
	10	0.6	4	839	0.401
	15	0.6	3	839	0.401
Oshodi	0	3	12	4194	0.459
	5	2	7	2796	0.304
	10	2	5	2796	0.28
	15	2	4	2796	0.21
Alimosho	0	22	80	30,795	0.502
	5	10	62	13,981	0.353
	10	8	62	11,185	0.335
	15	8	44	16,778	0.33
Sensitivity variable: Minimum battery state of charge					
Kosofe	30%	0.8	8	1392	0.511
Oshodi	30%	3	11	4194	0.441
Alimosho	30%	20	78	27,963	0.474
**Building Type: *‘Face ‘me ‘I ‘face ‘you’***					
Sensitivity variable: Maximum annual capacity shortage					
Kosofe	0	1.6	16	2237	0.538
	5	1.2	10	1678	0.44
	10	1	9	1398	0.425
	15	1	7	1398	0.421
Oshodi	0	6	36	8388	0.571
	5	3	24	4194	0.404
	10	3	18	4194	0.4
	15	3	14	4194	0.4
Alimosho	0	78	176	109,055	0.429
	5	34	164	47,537	0.279
	10	28	148	39,148	0.265
	15	28	98	39,148	0.26
Sensitivity variable: Minimum battery state of charge					
Kosofe	30%	1.6	4	2237	0.497
Oshodi	30%	4	40	5592	0.52
Alimosho	30%	70	176	97,870	0.399
**Building Type: Traditional court**					
Sensitivity variable: Maximum annual capacity shortage					
Kosofe	0	0.6	4	839	0.54
	5	0.4	3	559	0.452
	10	0.3	3	419	0.424
	15	0.3	3	419	0.424
Oshodi	0	6	30	8388	0.453
	5	4	12	5592	0.261
	10	3	12	4194	0.243
	15	3	8	4194	0.237
Alimosho	0	16	68	22,370	0.45
	5	8	56	11,185	0.32
	10	7	44	9787	0.304
	15	6	44	8389	0.294
Sensitivity variable: Minimum battery state of charge					
Kosofe	30%	0.5	4	699	0.504
Oshodi	30%	5	30	6990	0.416
Alimosho	30%	14	64	19,574	0.408
**Building Type: Flat Appartment**					
Sensitivity variable: Maximum annual capacity shortage					
Kosofe	0	3	18	4194	0.547
	5	2	14	2796	0.461
	10	2	10	2796	0.459
	15	2	10	2796	0.459
Oshodi	0	16	88	22,368	0.501
	5	8	74	11,184	0.387
	10	7	66	9786	0.376
	15	6	76	8388	0.376
Alimosho	0	42	76	58,772	0.743
	5	16	76	22,370	0.475
	10	12	74	16,778	0.436
	15	10	72	13,981	0.421
Sensitivity variable: Minimum battery state of charge					
Kosofe	30%	2	24	2796	0.529
Oshodi	30%	144	86	19,572	0.466
Alimosho	30%	36	74	50,333	0.678

**Table B3:** Effect of minimum battery state of charge and capacity shortage on PV system components and LCOE (minimum loads).

LGA	Sensitivity value (%)	PV array (kWh)	1 kWh Lead acid battery	PV power output (kWh/year)	LCOE
**Building Type: Duplex**					
Sensitivity variable: Maximum annual capacity shortage					
Kosofe	0	0.8	9	1118	0.552
	5	0.6	5	839	0.41
	10	0.6	4	839	0.401
	15	0.6	3	839	0.401
Oshodi	0	3	12	4194	0.459
	5	2	7	2796	0.304
	10	2	5	2796	0.28
	15	2	4	2796	0.21
Alimosho	0	22	80	30,795	0.502
	5	10	62	13,981	0.353
	10	8	62	11,185	0.335
	15	8	44	16,778	0.33
Sensitivity variable: Minimum battery state of charge					
Kosofe	30%	0.8	8	1392	0.511
Oshodi	30%	3	11	4194	0.441
Alimosho	30%	20	78	27,963	0.474
**Building Type: *‘Face- me -I –face- you’***					
Sensitivity variable: Maximum annual capacity shortage					
Kosofe	0	0.2	2	280	0.531
	5	0.2	1	280	0.391
	10	0.2	1	280	0.391
	15	0.1	1	140	0.322
Oshodi	0	2.5	22	3495	0.498
	5	1.5	18	3495	0.384
	10	1.5	12	2097	0.361
	15	1.5	9	2097	0.357
Alimosho	0	7	42	9787	0.422
	5	5	18	6991	0.253
	10	4	16	5593	0.223
	15	4	12	5593	0.219
Sensitivity variable: Minimum battery state of charge					
Kosofe	30%	0.3	1	419	0.472
Oshodi	30%	5.2	20	4395	0.47
Alimosho	30%	6	42	8389	0.395
**Building Type: Traditional Court**					
Sensitivity variable: Maximum annual capacity shortage					
Kosofe	0	0.3	2	419	0.575
	5	0.2	2	280	0.477
	10	0.2	1	280	0.463
	15	0.2	1	280	0.463
Oshodi	0	0.6	4	839	0.43
	5	0.5	2	699	0.284
	10	0.5	1	699	0.24
	15	0.4	1	559	0.23
Alimosho	0	1	8	1398	0.417
	5	0.8	4	1119	0.264
	10	0.8	2	1119	0.233
	15	0.6	3	839	0.214
Sensitivity variable: Minimum battery state of charge					
Kosofe	30%	0.3	2	419	0.554
Oshodi	30%	0.7	3	979	0.402
Alimosho	30%	1	7	1398	0.386
**Building Type: Flat Appartment**					
Sensitivity variable: Maximum annual capacity shortage					
Kosofe	0	0.7	3	979	0.449
	5	0.4	3	559	0.35
	10	0.3	3	419	0.336
	15	0.3	2	419	0.328
Oshodi	0	0.7	6	979	0.533
	5	0.4	5	559	0.412
	10	0.4	3	559	0.391
	15	0.4	3	559	0.383
Alimosho	0	5	22	6991	0.488
	5	3	14	4194	0.323
	10	2	20	2796	0.31
	15	2	12	2796	0.281
Sensitivity variable: Minimum battery state of charge					
Kosofe	30%	0.7	3	979	0.347
Oshodi	30%	0.7	5	979	0.481
Alimosho	30%	4	22	5593	0.401

**Table C4:** Effects of sensitivity variables on LCOE of PV systems Maximum Loads.

LGA	LCOE (5%DR & 2%IR, 25 years PV, 40% SOC)	LCOE (10% DR)	LCOE (5% IR)	LCOE (20 yrs PV lifetime	LCOE (30 yrs PV lifetime)	LCOE (30% SOC)
Duplex						
Kosofe	0.497	0.706	0.388	0.542	0.478	0.457
Oshodi	0.398	0.578	0.307	0.44	0.381	0.371
Alimosho	0.411	0.765	0.411	0.463	0.376	0.353
Single Family Bungalow						
Kosofe	0.508	0.702	0.407	0.541	0.495	0.463
Oshodi	0.452	0.648	0.349	0.495	0.434	0.416
Alimosho	0.513	0.718	0.406	0.553	0.497	0.495
*‘Face –me- I -face -you’*						
Kosofe	0.538	0.742	0.427	0.572	0.523	0.497
Oshodi	0.571	0.799	0.448	0.616	0.551	0.52
Alimosho	0.429	0.645	0.322	0.486	0.406	0.399
Traditional Court						
Kosofe	0.54	0.758	0.428	0.582	0.523	0.504
Oshodi	0.453	0.651	0.344	0.497	0.432	0.416
Alimosho	0.45	0.653	0.346	0.497	0.43	0.408
Flat Appartment						
Kosofe	0.547	0.763	0.435	0.591	0.529	0.529
Oshodi	0.501	0.717	0.391	0.547	0.482	0.466
Alimosho	0.743	$1.07	0.583	0.83	0.707	0.678

*Note*: $ = USD.

**Table C5:** Effects of sensitivity variables on LCOE of PV systems (Minimum Loads).

LGA	LCOE (5%DR & 2%IR, 25 years PV, 40% SOC)	LCOE (10% DR)	LCOE (5% IR)	LCOE (20 yrs PV lifetime	LCOE (30 yrs PV lifetime)	LCOE (30% SOC)
Duplex						
Kosofe	0.552	0.757	0.434	0.584	0.535	0.511
Oshodi	0.459	0.416	0.354	0.506	0.44	0.441
Alimosho	0.502	0.736	0.384	0.558	0.479	0.474
Single Family Bungalow						
Kosofe	0.529	0.755	0.414	0.578	0.509	0.493
Oshodi	0.439	0.639	0.339	0.486	0.42	0.413
Alimosho	0.432	0.634	0.332	0.477	0.413	0.406
*‘Face- me -I -face –you’*						
Kosofe	0.531	0.733	0.425	0.565	0.517	0.472
Oshodi	0.498	0.693	0.397	0.534	0.484	0.47
Alimosho	0.422	0.6	0.33	0.458	0.407	0.395
Traditional Court						
Kosofe	0.575	0.811	0.453	0.599	0.535	0.554
Oshodi	0.43	0.607	0.331	0.464	0.413	0.402
Alimosho	0.417	0.584	0.316	0.449	0.399	0.386
Flat Appartment						
Kosofe	0.449	0.651	0.347	0.494	0.431	0.347
Oshodi	0.533	0.743	0.419	0.571	0.517	0.481
Alimosho	0.488	0.649	0.346	0.494	0.429	0.401

## Appendix A

Table A1.

## Appendix B

Tables B2 and B3.

## Appendix C

Tables C4 and C5.

## Appendix D Supplementary materials

Image, application 1

Image, application 2
