# Supplementary material for: Data on the daily electricity load profile and solar photovoltaic (PV) system components for residential buildings in Lagos, Nigeria
Source: Data Brief. 2020 Apr 13;30:105531. doi: 10.1016/j.dib.2020.105531 (PMC7186491; doi:10.1016/j.dib.2020.105531)
Supplement: Supplementary file 1 [file mmc1.docx]

**QUESTIONNAIRE AND TIME OF USE DAIRY FOR DATA COLLECTION**

**Section 1: Socio-economic characteristics of Household**

- 1. Questionnaire Number:
  2. Street and House address:
  3. Occupation/profession of household head:

Farmer

Business woman/man

Retiree

Civil servant

Others……………………………………

**Section 2: Building Characteristics (please tick a box as appropriate)**

4. Which of the building type below best describes your dwelling/building?

Duplex

Single family bungalow

Flat/Apartment

Traditional court

‘*Face-me-i-face-you’*

Other, please specify_________________________

5. What is the total number of individuals (including children) living in your household? _____________

6. Please provide details on the following as applicable to your household

Number of bedrooms: _____ Number of lounge: _______ Number of toilets: ______

Number of dining room: _____ Number of Kitchen: ______

7. What is the gross external floor area^^[[1]](#footnote-1)^^ of your house? : ____ (m^2^)

8. Of what construction material is your dwelling composed of?

Brick

Concrete

Bamboo

Others  Please indicate: ­­­­­­­­­___________

9. Please provide information about the different alternative energy sources/lighting technologies in your household using the below table

| **Energy device** | **Tick if used** | **Usage frequency (tick more than one if applicable)** | **Average weekly quantity consumed** |
| --- | --- | --- | --- |
| Candle |  | Regularly  During grid outage only |  |
| Kerosene lamps |  | Regularly  During grid outage only |  |
| Diesel generators |  | Regularly  During grid outage only |  |
| Rechargeable lanterns |  | Regularly  During grid outage only | NA |
| Others: |  |  |  |

10. For which purpose is energy consumed most in your household?

Cooking

Heating the house

Lighting

Leisure (e.g. TV)

Others:  Please specify:­­­­_______________________________

**Section 3: Household energy consumption devices**

11. Please provide information about energy systems in your household using the table below.

| **Appliance** | **Tick if used** | **Quantity (Number)** | **Power rating (W)** | **Model (if power rating is unavailable)** |
| --- | --- | --- | --- | --- |
|  |  |  |  |  |
| Freezer |  |  |  |  |
| Fridge or fridge/freezer |  |  |  |  |
| Pressing iron |  |  |  |  |
| DVD (CD+video player) |  |  |  |  |
| Incandescent lamp |  |  |  |  |
| Fluorescent tube |  |  |  |  |
| Compact fluorescent lamp (CFL) |  |  |  |  |
| Light emitting diode (LED) |  |  |  |  |
| Television |  |  |  |  |
| Complete radio (simple radio+DVD) |  |  |  |  |
| Stereo speaker |  |  |  |  |
| Laptop |  |  |  |  |
| Electrical water coil |  |  |  |  |
| Computer |  |  |  |  |
| Fan |  |  |  |  |
| Vacuum cleaner |  |  |  |  |
| Air conditioner |  |  |  |  |
| Heater (for space heating) |  |  |  |  |
| Micro wave |  |  |  |  |
| Gas and electric oven |  |  |  |  |
| Food blender/mixer |  |  |  |  |
| Coffee maker |  |  |  |  |
| Washing machine |  |  |  |  |
| Shaving Machine |  |  |  |  |
| Voltage regulator |  |  |  |  |
| Wood |  | Kg: |  |  |
| Others |  |  |  |  |

**Section 4: Household energy consumption pattern**

12. For each of the appliance used in your household (in section 3) and for the different days of the week, please provide information on the duration of hours used and the period of the day during which it is used. Please see the example below

**Example:** Last Monday, Jonathan’s security light (fluorescent bulb) was on from 6 pm to midnight and he charged his mobile phone from 8 pm to 10 pm.

| **Appliance** | **Duration of use (hours)** | **Time of the day** | | | | | | | | | | | | | | | | | | | | | | | |
| --- | --- | --- | --- | --- | --- | --- | --- | --- | --- | --- | --- | --- | --- | --- | --- | --- | --- | --- | --- | --- | --- | --- | --- | --- | --- |
|  |  | **6am** | **7** | **8** | **9** | **10** | **11** | **12** | **1pm** | **2** | **3** | **4** | **5** | **6** | **7** | **8** | **9** | **10** | **11** | **00** | **1am** | **2** | **3** | **4** | **5** |
| Fluorescent bulb | 6 |  |  |  |  |  |  |  |  |  |  |  |  |  |  |  |  |  |  |  |  |  |  |  |  |
| Mobile phone charger | **2** |  |  |  |  |  |  |  |  |  |  |  |  |  |  |  |  |  |  |  |  |  |  |  |  |
|  |  |  |  |  |  |  |  |  |  |  |  |  |  |  |  |  |  |  |  |  |  |  |  |  |  |

**Monday**

| **Appliance** | **Duration of use (hours)** | **Time of the day** | | | | | | | | | | | | | | | | | | | | | | | |
| --- | --- | --- | --- | --- | --- | --- | --- | --- | --- | --- | --- | --- | --- | --- | --- | --- | --- | --- | --- | --- | --- | --- | --- | --- | --- |
|  |  | **6am** | **7** | **8** | **9** | **10** | **11** | **12** | **1pm** | **2** | **3** | **4** | **5** | **6** | **7** | **8** | **9** | **10** | **11** | **00** | **1am** | **2** | **3** | **4** | **5** |
|  |  |  |  |  |  |  |  |  |  |  |  |  |  |  |  |  |  |  |  |  |  |  |  |  |  |
|  |  |  |  |  |  |  |  |  |  |  |  |  |  |  |  |  |  |  |  |  |  |  |  |  |  |
|  |  |  |  |  |  |  |  |  |  |  |  |  |  |  |  |  |  |  |  |  |  |  |  |  |  |
|  |  |  |  |  |  |  |  |  |  |  |  |  |  |  |  |  |  |  |  |  |  |  |  |  |  |
|  |  |  |  |  |  |  |  |  |  |  |  |  |  |  |  |  |  |  |  |  |  |  |  |  |  |
|  |  |  |  |  |  |  |  |  |  |  |  |  |  |  |  |  |  |  |  |  |  |  |  |  |  |
|  |  |  |  |  |  |  |  |  |  |  |  |  |  |  |  |  |  |  |  |  |  |  |  |  |  |
|  |  |  |  |  |  |  |  |  |  |  |  |  |  |  |  |  |  |  |  |  |  |  |  |  |  |
|  |  |  |  |  |  |  |  |  |  |  |  |  |  |  |  |  |  |  |  |  |  |  |  |  |  |
|  |  |  |  |  |  |  |  |  |  |  |  |  |  |  |  |  |  |  |  |  |  |  |  |  |  |
|  |  |  |  |  |  |  |  |  |  |  |  |  |  |  |  |  |  |  |  |  |  |  |  |  |  |
|  |  |  |  |  |  |  |  |  |  |  |  |  |  |  |  |  |  |  |  |  |  |  |  |  |  |
|  |  |  |  |  |  |  |  |  |  |  |  |  |  |  |  |  |  |  |  |  |  |  |  |  |  |

**Tuesday**

| **Appliance** | **Duration of use (hours)** | **Time of the day** | | | | | | | | | | | | | | | | | | | | | | | |
| --- | --- | --- | --- | --- | --- | --- | --- | --- | --- | --- | --- | --- | --- | --- | --- | --- | --- | --- | --- | --- | --- | --- | --- | --- | --- |
|  |  | **6am** | **7** | **8** | **9** | **10** | **11** | **12** | **1pm** | **2** | **3** | **4** | **5** | **6** | **7** | **8** | **9** | **10** | **11** | **00** | **1am** | **2** | **3** | **4** | **5** |
|  |  |  |  |  |  |  |  |  |  |  |  |  |  |  |  |  |  |  |  |  |  |  |  |  |  |
|  |  |  |  |  |  |  |  |  |  |  |  |  |  |  |  |  |  |  |  |  |  |  |  |  |  |
|  |  |  |  |  |  |  |  |  |  |  |  |  |  |  |  |  |  |  |  |  |  |  |  |  |  |
|  |  |  |  |  |  |  |  |  |  |  |  |  |  |  |  |  |  |  |  |  |  |  |  |  |  |
|  |  |  |  |  |  |  |  |  |  |  |  |  |  |  |  |  |  |  |  |  |  |  |  |  |  |
|  |  |  |  |  |  |  |  |  |  |  |  |  |  |  |  |  |  |  |  |  |  |  |  |  |  |
|  |  |  |  |  |  |  |  |  |  |  |  |  |  |  |  |  |  |  |  |  |  |  |  |  |  |
|  |  |  |  |  |  |  |  |  |  |  |  |  |  |  |  |  |  |  |  |  |  |  |  |  |  |
|  |  |  |  |  |  |  |  |  |  |  |  |  |  |  |  |  |  |  |  |  |  |  |  |  |  |
|  |  |  |  |  |  |  |  |  |  |  |  |  |  |  |  |  |  |  |  |  |  |  |  |  |  |
|  |  |  |  |  |  |  |  |  |  |  |  |  |  |  |  |  |  |  |  |  |  |  |  |  |  |
|  |  |  |  |  |  |  |  |  |  |  |  |  |  |  |  |  |  |  |  |  |  |  |  |  |  |
|  |  |  |  |  |  |  |  |  |  |  |  |  |  |  |  |  |  |  |  |  |  |  |  |  |  |
|  |  |  |  |  |  |  |  |  |  |  |  |  |  |  |  |  |  |  |  |  |  |  |  |  |  |

**Wednesday**

| **Appliance** | **Duration of use (hours)** | **Time of the day** | | | | | | | | | | | | | | | | | | | | | | | |
| --- | --- | --- | --- | --- | --- | --- | --- | --- | --- | --- | --- | --- | --- | --- | --- | --- | --- | --- | --- | --- | --- | --- | --- | --- | --- |
|  |  | **6am** | **7** | **8** | **9** | **10** | **11** | **12** | **1pm** | **2** | **3** | **4** | **5** | **6** | **7** | **8** | **9** | **10** | **11** | **00** | **1am** | **2** | **3** | **4** | **5** |
|  |  |  |  |  |  |  |  |  |  |  |  |  |  |  |  |  |  |  |  |  |  |  |  |  |  |
|  |  |  |  |  |  |  |  |  |  |  |  |  |  |  |  |  |  |  |  |  |  |  |  |  |  |
|  |  |  |  |  |  |  |  |  |  |  |  |  |  |  |  |  |  |  |  |  |  |  |  |  |  |
|  |  |  |  |  |  |  |  |  |  |  |  |  |  |  |  |  |  |  |  |  |  |  |  |  |  |
|  |  |  |  |  |  |  |  |  |  |  |  |  |  |  |  |  |  |  |  |  |  |  |  |  |  |
|  |  |  |  |  |  |  |  |  |  |  |  |  |  |  |  |  |  |  |  |  |  |  |  |  |  |
|  |  |  |  |  |  |  |  |  |  |  |  |  |  |  |  |  |  |  |  |  |  |  |  |  |  |
|  |  |  |  |  |  |  |  |  |  |  |  |  |  |  |  |  |  |  |  |  |  |  |  |  |  |
|  |  |  |  |  |  |  |  |  |  |  |  |  |  |  |  |  |  |  |  |  |  |  |  |  |  |
|  |  |  |  |  |  |  |  |  |  |  |  |  |  |  |  |  |  |  |  |  |  |  |  |  |  |
|  |  |  |  |  |  |  |  |  |  |  |  |  |  |  |  |  |  |  |  |  |  |  |  |  |  |
|  |  |  |  |  |  |  |  |  |  |  |  |  |  |  |  |  |  |  |  |  |  |  |  |  |  |
|  |  |  |  |  |  |  |  |  |  |  |  |  |  |  |  |  |  |  |  |  |  |  |  |  |  |
|  |  |  |  |  |  |  |  |  |  |  |  |  |  |  |  |  |  |  |  |  |  |  |  |  |  |

**Thursday**

| **Appliance** | **Duration of use (hours)** | **Time of the day** | | | | | | | | | | | | | | | | | | | | | | | |
| --- | --- | --- | --- | --- | --- | --- | --- | --- | --- | --- | --- | --- | --- | --- | --- | --- | --- | --- | --- | --- | --- | --- | --- | --- | --- |
|  |  | **6am** | **7** | **8** | **9** | **10** | **11** | **12** | **1pm** | **2** | **3** | **4** | **5** | **6** | **7** | **8** | **9** | **10** | **11** | **00** | **1am** | **2** | **3** | **4** | **5** |
|  |  |  |  |  |  |  |  |  |  |  |  |  |  |  |  |  |  |  |  |  |  |  |  |  |  |
|  |  |  |  |  |  |  |  |  |  |  |  |  |  |  |  |  |  |  |  |  |  |  |  |  |  |
|  |  |  |  |  |  |  |  |  |  |  |  |  |  |  |  |  |  |  |  |  |  |  |  |  |  |
|  |  |  |  |  |  |  |  |  |  |  |  |  |  |  |  |  |  |  |  |  |  |  |  |  |  |
|  |  |  |  |  |  |  |  |  |  |  |  |  |  |  |  |  |  |  |  |  |  |  |  |  |  |
|  |  |  |  |  |  |  |  |  |  |  |  |  |  |  |  |  |  |  |  |  |  |  |  |  |  |
|  |  |  |  |  |  |  |  |  |  |  |  |  |  |  |  |  |  |  |  |  |  |  |  |  |  |
|  |  |  |  |  |  |  |  |  |  |  |  |  |  |  |  |  |  |  |  |  |  |  |  |  |  |
|  |  |  |  |  |  |  |  |  |  |  |  |  |  |  |  |  |  |  |  |  |  |  |  |  |  |
|  |  |  |  |  |  |  |  |  |  |  |  |  |  |  |  |  |  |  |  |  |  |  |  |  |  |
|  |  |  |  |  |  |  |  |  |  |  |  |  |  |  |  |  |  |  |  |  |  |  |  |  |  |
|  |  |  |  |  |  |  |  |  |  |  |  |  |  |  |  |  |  |  |  |  |  |  |  |  |  |
|  |  |  |  |  |  |  |  |  |  |  |  |  |  |  |  |  |  |  |  |  |  |  |  |  |  |
|  |  |  |  |  |  |  |  |  |  |  |  |  |  |  |  |  |  |  |  |  |  |  |  |  |  |

**Friday**

| **Appliance** | **Duration of use (hours)** | **Time of the day** | | | | | | | | | | | | | | | | | | | | | | | |
| --- | --- | --- | --- | --- | --- | --- | --- | --- | --- | --- | --- | --- | --- | --- | --- | --- | --- | --- | --- | --- | --- | --- | --- | --- | --- |
|  |  | **6am** | **7** | **8** | **9** | **10** | **11** | **12** | **1pm** | **2** | **3** | **4** | **5** | **6** | **7** | **8** | **9** | **10** | **11** | **00** | **1am** | **2** | **3** | **4** | **5** |
|  |  |  |  |  |  |  |  |  |  |  |  |  |  |  |  |  |  |  |  |  |  |  |  |  |  |
|  |  |  |  |  |  |  |  |  |  |  |  |  |  |  |  |  |  |  |  |  |  |  |  |  |  |
|  |  |  |  |  |  |  |  |  |  |  |  |  |  |  |  |  |  |  |  |  |  |  |  |  |  |
|  |  |  |  |  |  |  |  |  |  |  |  |  |  |  |  |  |  |  |  |  |  |  |  |  |  |
|  |  |  |  |  |  |  |  |  |  |  |  |  |  |  |  |  |  |  |  |  |  |  |  |  |  |
|  |  |  |  |  |  |  |  |  |  |  |  |  |  |  |  |  |  |  |  |  |  |  |  |  |  |
|  |  |  |  |  |  |  |  |  |  |  |  |  |  |  |  |  |  |  |  |  |  |  |  |  |  |
|  |  |  |  |  |  |  |  |  |  |  |  |  |  |  |  |  |  |  |  |  |  |  |  |  |  |
|  |  |  |  |  |  |  |  |  |  |  |  |  |  |  |  |  |  |  |  |  |  |  |  |  |  |
|  |  |  |  |  |  |  |  |  |  |  |  |  |  |  |  |  |  |  |  |  |  |  |  |  |  |
|  |  |  |  |  |  |  |  |  |  |  |  |  |  |  |  |  |  |  |  |  |  |  |  |  |  |
|  |  |  |  |  |  |  |  |  |  |  |  |  |  |  |  |  |  |  |  |  |  |  |  |  |  |
|  |  |  |  |  |  |  |  |  |  |  |  |  |  |  |  |  |  |  |  |  |  |  |  |  |  |
|  |  |  |  |  |  |  |  |  |  |  |  |  |  |  |  |  |  |  |  |  |  |  |  |  |  |

**Saturday**

| **Appliance** | **Duration of use (hours)** | **Time of the day** | | | | | | | | | | | | | | | | | | | | | | | |
| --- | --- | --- | --- | --- | --- | --- | --- | --- | --- | --- | --- | --- | --- | --- | --- | --- | --- | --- | --- | --- | --- | --- | --- | --- | --- |
|  |  | **6am** | **7** | **8** | **9** | **10** | **11** | **12** | **1pm** | **2** | **3** | **4** | **5** | **6** | **7** | **8** | **9** | **10** | **11** | **00** | **1am** | **2** | **3** | **4** | **5** |
|  |  |  |  |  |  |  |  |  |  |  |  |  |  |  |  |  |  |  |  |  |  |  |  |  |  |
|  |  |  |  |  |  |  |  |  |  |  |  |  |  |  |  |  |  |  |  |  |  |  |  |  |  |
|  |  |  |  |  |  |  |  |  |  |  |  |  |  |  |  |  |  |  |  |  |  |  |  |  |  |
|  |  |  |  |  |  |  |  |  |  |  |  |  |  |  |  |  |  |  |  |  |  |  |  |  |  |
|  |  |  |  |  |  |  |  |  |  |  |  |  |  |  |  |  |  |  |  |  |  |  |  |  |  |
|  |  |  |  |  |  |  |  |  |  |  |  |  |  |  |  |  |  |  |  |  |  |  |  |  |  |
|  |  |  |  |  |  |  |  |  |  |  |  |  |  |  |  |  |  |  |  |  |  |  |  |  |  |
|  |  |  |  |  |  |  |  |  |  |  |  |  |  |  |  |  |  |  |  |  |  |  |  |  |  |
|  |  |  |  |  |  |  |  |  |  |  |  |  |  |  |  |  |  |  |  |  |  |  |  |  |  |
|  |  |  |  |  |  |  |  |  |  |  |  |  |  |  |  |  |  |  |  |  |  |  |  |  |  |
|  |  |  |  |  |  |  |  |  |  |  |  |  |  |  |  |  |  |  |  |  |  |  |  |  |  |
|  |  |  |  |  |  |  |  |  |  |  |  |  |  |  |  |  |  |  |  |  |  |  |  |  |  |
|  |  |  |  |  |  |  |  |  |  |  |  |  |  |  |  |  |  |  |  |  |  |  |  |  |  |
|  |  |  |  |  |  |  |  |  |  |  |  |  |  |  |  |  |  |  |  |  |  |  |  |  |  |

**Sunday**

| **Appliance** | **Duration of use (hours)** | **Time of the day** | | | | | | | | | | | | | | | | | | | | | | | |
| --- | --- | --- | --- | --- | --- | --- | --- | --- | --- | --- | --- | --- | --- | --- | --- | --- | --- | --- | --- | --- | --- | --- | --- | --- | --- |
|  |  | **6am** | **7** | **8** | **9** | **10** | **11** | **12** | **1pm** | **2** | **3** | **4** | **5** | **6** | **7** | **8** | **9** | **10** | **11** | **00** | **1am** | **2** | **3** | **4** | **5** |
|  |  |  |  |  |  |  |  |  |  |  |  |  |  |  |  |  |  |  |  |  |  |  |  |  |  |
|  |  |  |  |  |  |  |  |  |  |  |  |  |  |  |  |  |  |  |  |  |  |  |  |  |  |
|  |  |  |  |  |  |  |  |  |  |  |  |  |  |  |  |  |  |  |  |  |  |  |  |  |  |
|  |  |  |  |  |  |  |  |  |  |  |  |  |  |  |  |  |  |  |  |  |  |  |  |  |  |
|  |  |  |  |  |  |  |  |  |  |  |  |  |  |  |  |  |  |  |  |  |  |  |  |  |  |
|  |  |  |  |  |  |  |  |  |  |  |  |  |  |  |  |  |  |  |  |  |  |  |  |  |  |
|  |  |  |  |  |  |  |  |  |  |  |  |  |  |  |  |  |  |  |  |  |  |  |  |  |  |
|  |  |  |  |  |  |  |  |  |  |  |  |  |  |  |  |  |  |  |  |  |  |  |  |  |  |
|  |  |  |  |  |  |  |  |  |  |  |  |  |  |  |  |  |  |  |  |  |  |  |  |  |  |
|  |  |  |  |  |  |  |  |  |  |  |  |  |  |  |  |  |  |  |  |  |  |  |  |  |  |
|  |  |  |  |  |  |  |  |  |  |  |  |  |  |  |  |  |  |  |  |  |  |  |  |  |  |
|  |  |  |  |  |  |  |  |  |  |  |  |  |  |  |  |  |  |  |  |  |  |  |  |  |  |

**THANK YOU FOR YOUR COOPERATION**

1. The whole area of the building, including external walls [↑](#footnote-ref-1)
